# Association of Timing of Sexual Partnerships and Perceptions of Partners' Concurrency With Reporting of Sexually Transmitted Infection Diagnosis

**DOI:** 10.1001/jamanetworkopen.2018.5957

**Published:** 2018-12-14

**Authors:** Catherine H. Mercer, Kyle G. Jones, Rebecca S. Geary, Nigel Field, Clare Tanton, Sarah Burkill, Soazig Clifton, Pam Sonnenberg, Kirstin R. Mitchell, Kirsten Gravningen, Anne M. Johnson

**Affiliations:** 1 Institute for Global Health, University College London, London, United Kingdom; 2 Department of Health Services Research and Policy, London School of Hygiene and Tropical Medicine, London, United Kingdom; 3 Medical Research Council Tropical Epidemiology Group, London School of Hygiene and Tropical Medicine, London, United Kingdom; 4 Medical Research Council/Chief Scientist Office, Social and Public Health Sciences Unit, University of Glasgow, Glasgow, United Kingdom; 5 Department of Microbiology and Infection Control, University Hospital of North Norway, Tromsø, Norway

## Abstract

**Importance:**

The timing of sexual partnerships is important for sexually transmitted infection (STI) transmission potential. Studies often measure timing as whether partnerships overlap in time (concurrency), but this measure does not account for how STI risk from previous partners can be carried forward into future partnerships even when there is a time gap between them (serial monogamy) if the infectious period is greater than this time gap.

**Objective:**

To examine the association of the timing of partnerships, measured as the time gap or time overlap between partners, and perceptions of partners’ concurrency with STI transmission.

**Design, Setting, and Participants:**

This survey study that was conducted in 2017 included 8867 participants in Britain aged 16 to 44 years who reported 1 or more sexual partners in the 5 years before the interview. Data were collected from 2010 to 2012 from Britain’s third National Survey of Sexual Attitudes and Lifestyles (Natsal-3), a large probability survey (response rate, 57.7%) designed to be broadly representative of the general population.

**Exposures:**

Gaps between participants’ 3 or fewer most recent partners in the past 5 years were calculated from dates of the last sexual encounter with former partners and the first sexual encounter with subsequent partners. Negative gaps denote overlapping partnerships (concurrency); positive gaps denote serial monogamy. Participant perception of most recent partner concurrency was proxied by asking participants whether they knew or thought that their partners had had sex with other partners since their first sexual encounter together.

**Main Outcomes and Measures:**

Reported STI diagnosis in the past 5 years.

**Results:**

Of 8867 participants eligible for this analysis, 3509 (39.6%) were male and 5158 (58.2%) were female, with a mean age of 28 years. Overall, 48.1% of males and 39.5% of females reported 2 or more partners and 1 or more time gaps. The median time gap was 2 months (interquartile range, −3 months to 8 months). Although 67.0% of the time gaps were 1 month or more, many were sufficiently short time gaps for STI transmission. The time gap was independently associated with STI diagnosis, without a significant decrease in likelihood until the time gap was 4 months or more for females (adjusted odds ratio [OR]: 0.39, 95% CI, 0.19-0.81) and 6 months or more for males (adjusted OR: 0.42, 95% CI, 0.20-0.85) compared with time overlaps of 2 years or more. Participant perception of partners’ concurrency (reported by half of the participants) was independently associated with STI diagnosis among females (reporting no partner concurrency vs reporting partner concurrency: adjusted OR, 0.32; 95% CI, 0.22-0.49).

**Conclusions and Relevance:**

The findings suggest that the gap between partners is often sufficiently small to permit STI transmission and that many people, although themselves monogamous, have partners who are not, which itself is associated with an increase in the risk of STI acquisition. Public health practitioners should communicate these epidemiological facts, and researchers should develop measures that better capture the risk of STI transmission from partners.

## Key Points

**Question:**

What is the association of the timing of sexual partnerships, measured as the time gap (serial monogamy) and perceived time overlap (concurrency) among most recent partners, with sexually transmitted infection diagnosis?

**Findings:**

In this survey study of 8867 sexually active people aged 16 to 44 years living in Britain, 61.1% of men and 64.8% of women with multiple partners were serially monogamous. The likelihood of sexually transmitted infection diagnosis decreased significantly when the time gap between partners was 4 months or more for females and 6 months or more for males, and sexually transmitted infection diagnoses were more common among the half of women who knew or perceived that their partners had other partners independent of the number of, or gap between, their partners.

**Meaning:**

The findings suggest that commonly held assumptions that sexually transmitted infection transmission cannot occur if partners are serially monogamous need to be challenged because short gaps between partners appear to be important for sexually transmitted infection transmission.

## Teaser

This survey study of British individuals examines the association of the timing of sexual partnerships, measured as time gaps (serial monogamy) and perceived time overlaps (concurrency) among most recent partners, with sexually transmitted infection diagnosis.

## Introduction

The number of sexual partners that individuals have is widely recognized as a key indicator of their likelihood of acquiring a sexually transmitted infection (STI).^1^ In addition, the timing of partnerships in terms of whether they overlap in time (concurrency) may further be associated with increased risk of STI transmission because concurrency may increase partner change rates and exposure to additional risk of STIs from the partner’s partners.^1,2,3,4^ Many studies consider concurrency as a dichotomous behavior, but doing so overlooks how the risk from previous partners can be carried forward into future partnerships even if there is a time gap between them (serial monogamy) when the infectious period is greater than this gap. For example, the infectious period is weeks for some STIs (eg, an estimated 6-8 weeks for infection due to *Neisseria gonorrheae*) but longer for others (eg, 12-18 months for infection due to *Chlamydia trachomatis*)^5,6^; thus, individuals may be able to spread infection to new partners after their previous partnerships have ended. Because many STIs are asymptomatic, infected individuals may not recognize a need to test for STIs at the end of a relationship or use condoms with a new sexual partner. Consideration of the length of the time gap between partners, not just whether there is a time overlap, may therefore expand our understanding of the epidemiological characteristics of STI transmission and be important for informing effective STI prevention.

Risk of STI is associated with not only the partner’s characteristics and behavior but also those of the partner’s partners and their sexual networks.^7^ Taking these associations into account is epidemiologically important, but doing so in practice is difficult, which may in part explain why partners’ behavior and concurrency have received little attention. Studies that have sought to examine these associations have typically relied on individuals to report their partners’ characteristics and behaviors, such as whether individuals think their partners have had other partners since their first sex encounter together.^8,9,10,11,12^ Studies that have also obtained data firsthand from the partner have found poor agreement between the 2 accounts.^8,9,12^ However, an individual’s perception of their partner’s concurrency has been shown to be associated with increased STI risk independent of their own concurrent behavior.^8,10,11^ If an individual thinks their partner has other partner(s), this opinion may influence their decision making regarding, for example, condom use with the partner or subsequent partners; the timing of first sex with a subsequent partner; STI testing behavior; and ultimately, the likelihood of STI transmission.

This study used data representative of the British population to describe recent partnership histories, focusing on the timing of partnerships in terms of the time gap or overlap between partners and perceptions of partners’ concurrency, and to examine how these factors are associated with STI diagnosis.

## Methods

This survey study followed the American Association for Public Opinion Research (AAPOR) reporting guidelines. Data were analyzed from the third National Survey of Sexual Attitudes and Lifestyles (Natsal-3), a stratified probability sample survey undertaken from 2010 to 2012 of 15 162 people aged 16 to 74 years who resided in Britain. Natsal-3 was approved by the Oxfordshire Research Ethics Committee A, and participants provided oral informed consent. The present study did not require institutional review board approval because it used data from Natsal-3.The overall response rate was 57.7% (of all addresses known or estimated to be eligible according to the American Association for Public Opinion Research Response Rate 3 calculation^13^), consistent with other major social surveys undertaken contemporaneously.^14^ Natsal-3 is a collaboration among University College London (London, United Kingdom), the London School of Hygiene and Tropical Medicine (London), NatCen Social Research (London), Public Health England (formerly the Health Protection Agency) (London), and the University of Manchester (Manchester, United Kingdom). Full details of the Natsal-3 methods, including the questionnaire, have been published elsewhere.^14,15^

Natsal-3 used a combination of computer-assisted personal interviewing with computer-assisted self-interview (CASI) for the more sensitive questions, including those about participants’ sexual partners (defined as people who have had sex together—whether just once or a few times or as regular partners or married partners; having sex together was defined as vaginal, oral, and anal sexual intercourse). The CASI included a module on participants’ 3 or fewer most recent partners in the 5 years before the interview (referred to as the most recent partner module).^16^ If participants reported 2 or more most recent partners in the past 5 years, this module was repeated for their second most recent partner and, if applicable, third most recent partner during this period. This module included questions about the month and year of the first sexual encounter and most recent sexual encounter with these partners from which the time gap in months between the last sexual encounter with a former most recent partner and first sexual encounter with the subsequent most recent partner was calculated. Thus, 1 time gap could be calculated for participants reporting 2 most recent partners and 2 time gaps for those reporting 3 most recent partners. Examples of how the time gap was calculated are given in the eFigure in the Supplement.

Defining the start and end of partnerships assumes that these events are well defined, which is not always the case.^17^ In addition, this method does not allow for partnership reformation (eg, the first sexual encounter with a most recent partner occurred many months or years previously, and the partnership ended but then reformed later) because this appears as 1 continuous partnership in the data. If other partnerships occurred in the interim, the method would result in long negative time gaps (time overlaps), implying concurrency occurred even though the original partnership had (at that time) ended. As such, the date of the first sexual encounter between partners for negative time gaps (time overlaps) of more than 5 years was adjusted so that the length of these time gaps was truncated to a maximum of 5 years.

All sexually active participants were categorized according to their partnership history in the 5 years before the interview (hereafter referred to as recent partnership history), defined according to the number of most recent partners that they reported (1, 2, or 3), and for participants reporting 2 or more most recent partners, the timing of their most recent partnerships: concurrent partnerships (negative time gaps or time overlaps), serial monogamy (positive time gaps), or unknown (time gaps of 0 months or missing time data).

Participant perception of their partners’ concurrency was captured through a question in the most recent partner module that asked whether they thought that their most recent partner(s) had had sex with other sexual partner(s) since they first had sex together (for partnerships in which sex had occurred more than once; ie, concurrently during their relationship), with response options of yes, probably, probably not, no, and prefer not to say.

For this study’s outcome, self-reported data were used from the CASI on whether participants had ever received an STI diagnosis and, if so, when, from which we derived a binary variable for reporting STI diagnosis in the 5 years before the interview (hereafter referred to as STI diagnosis).

Because of the low partner change rates among those 45 years and older^15^ and, relatedly, the low STI prevalence among older individuals,^18^ the denominator of this study was limited to participants aged 16 to 44 years and specifically those sexually active in the 5 years before the interview (91.9%; 95% CI, 91.3%-92.5%; no significant sex difference) and those who reported data on 1 or more recent partner (98.4% of sexually active participants).

### Statistical Analysis

Stata version 13 (StataCorp) was used for complex survey analyses to account for the stratification, clustering, and weighting of the data.^14,15^ The demographic characteristics of participants eligible for this study were first analyzed. Participants’ recent partnership histories were examined according to the number and timing of their most recent partners in the past 5 years in terms of whether the dates of these partnerships suggested they had occurred concurrently or serial monogamously (or whether their timing was unknown), and the resulting percentage distributions were compared by age group.

The data were transposed for participants reporting 2 or more most recent partners so that each row of data then corresponded to either the time gap between their second most recent partner and first most recent partner or the gap between their third and second most recent partners (where applicable). The cumulative distribution of gaps by age group for each sex was examined and summarized using the median and interquartile range (IQR).

The participant-level data set was used to examine whether STI diagnosis varied according to the time gap between most recent partners (using the shorter, more negative gap or overlap if there were data on 2 time gaps). The time gap was categorized with a balance between categories that were sufficiently nuanced to be meaningful (including distinctions for clinical practice [eg, sexual history taking]^19^) and reflecting the goal of moving away from a concurrency or serial monogamy dichotomy; however, the gap categories were pragmatic in terms of sample size, especially given the low population prevalence of STI diagnosis.^18^ In addition to considering this measure of the participant’s own concurrency, we sought to assess whether any association with STI diagnosis remained statistically significant after taking into account participants’ perceptions of their partners’ concurrency (allowing responses of yes or probably to trump probably not or no when ≥2 most recent partners were reported). Using multivariable logistic regression also allowed for an accounting of the potentially confounding effects of participant age and reported partner numbers.^18^ The Pearson χ^2^ statistic (adapted for complex surveys) was used to determine the strength of evidence against the null hypothesis. A 2-sided *P* value of less than .05 was considered to be statistically significant. Participants with missing data were excluded from analyses because item nonresponse was low in Natsal-3 (typically <3%),^15^ including nonresponse to the questions in the most recent partners module. Finally, all analyses were presented separately by sex owing to differences in the experience and reporting of sexual behaviors^15^ and the sexual scripts that shape these behaviors.^20^

## Results

### Characteristics of the Study Sample

Of 8867 participants eligible for this analysis, 3509 (39.6%) were male and 5158 (58.2%) were female, with a mean age of 28 years (range, 16 to 44 years). All participants reported 1 or more partners in the 5 years before the interview and 1 or more most recent partners in the most recent partner module. These numbers corresponded to a weighted sample of 3568 males and 3585 females, on which the following estimates were based.

Age was evenly distributed across the sample age range and was similarly distributed for males and females (eTable 1 in the Supplement). More than half of the sample (men, 58.9%; women, 62.5%) reported living with a partner at the interview; 16.7% of participants were in steady relationships, whereas 24.4% of male participants and 20.8% of female participants were not. Participants predominantly identified as heterosexual or straight (96.9% of male participants; 96.0% of female participants) and of British white or non-British white race/ethnicity (85.7% of male participants; 86.8% of female participants). More than half of the sample (62.3% of male participants; 54.1% of female participants) reported that they did not belong to a religion. In terms of socioeconomic markers, more than half of the sample had achieved or were studying for higher academic qualifications (54.1% of male participants; 56.6% of female participants), one-third of the sample had managerial or professional occupations (34.7% of male participants and 32.7% of female participants),^21^ and the sample was evenly distributed in terms of area-level deprivation.^22^

### Recent Partnership Histories

Overall, 51.9% (95% CI, 49.9%-53.9%) of sexually active males and 60.5% (95% CI, 58.9%-62.0%) of sexually active females reported only 1 partner in the past 5 years (Table 1). Overall, 14.9% (95% CI, 13.6%-16.4%) of males and 14.8% (95% CI, 13.8%-15.8%) of females reported 2 partners during this time, with 66.5% (95% CI, 63.4%-69.5%) of these individuals (no significant sex difference) having their most recent partners serially monogamously (ie, the dates of these partnerships did not overlap). A total of 33.2% of males (95% CI, 31.4%-35.0%) and 24.8% of females (95% CI, 23.4%-26.2%) reported data on their 3 most recent partners, and serial monogamy was as likely as concurrency according to partnership dates. In total, 18.7% (95% CI, 17.2%-20.2%) of all sexually active males and 13.9% (95% CI, 12.9%-15.0%) of all sexually active females had concurrent partnerships in the past 5 years according to the dates of their 3 most recent partners.

**Table 1.** Past 5-Year Partnership Histories by Sex and Age Group Among Sexually Active Participants Aged 16 to 44 Years in Britain, 2010-2012^a^

Variable	Participant Age Group							
	Overall		16-24 y		25-34 y		35-44 y	
	% (95% CI)	Those With 2-3 Partners, %	% (95% CI)	Those With 2-3 Partners, %	% (95% CI)	Those With 2-3 Partners, %	% (95% CI)	Those With 2-3 Partners, %
**Males**								
1 Partner in past 5 y	51.9 (49.9-53.9)	NA	26.0 (23.5-28.6)	NA	50.7 (47.8-53.6)	NA	72.3 (68.7-75.6)	NA
2 Partners in past 5 y	14.9 (13.6-16.4)	100	17.1 (14.9-19.7)	100	15.8 (13.9-18.0)	100	12.5 (10.2-15.2)	100
Time overlap (concurrency)	4.0 (3.3-4.9)	27.0	2.1 (1.4-3.2)	12.5	4.6 (3.5-6.0)	29.1	4.9 (3.4-7.)	39.4
Time gap (serially monogamous)	9.7 (8.6-10.8)	64.6	13.9 (11.8-16.3)	81.3	9.7 (8.2-11.5)	61.4	6.4 (4.8-8.4)	51.4
Timing unknown	1.3 (0.9-1.7)	8.4	1.1 (0.7-1.7)	6.3	1.5 (0.9-2.4)	9.5	1.2 (0.6-2.1)	9.2
3 Partners in past 5 y	33.2 (31.4-35.0)	100	56.9 (53.9-59.8)	100	33.5 (30.8-36.3)	100	15.3 (12.7-18.2)	100
Both time overlaps (concurrent)	2.5 (2.0-3.1)	7.5	3.0 (2.0-4.5)	5.3	2.7 (2.0-3.7)	8.1	1.9 (1.1-3.3)	12.4
Both time gaps (serially monogamous)	13.6 (12.4-14.9)	40.9	27.8 (25.2-30.5)	48.9	12.1 (10.3-14.1)	36.1	4.4 (3.1-6.3)	29.0
1 Time overlap (concurrent), 1 time gap (serially monogamous)	12.2 (11.0-13.4)	36.6	17.1 (15.0-19.4)	30.0	13.3 (11.4-15.4)	39.7	7.4 (5.6-9.7)	48.4
Timing unknown	5.0 (4.2-5.8)	15.0	9.0 (7.4-10.9)	15.9	5.4 (4.2-6.8)	16.1	1.6 (0.9-2.7)	10.2
Any concurrency^b^	18.7 (17.2-20.2)	NA	22.2 (19.8-24.8)	NA	20.6 (18.4-23.0)	NA	14.2 (11.6-17.2)	NA
Denominators, unweighted/weighted	3509/3568	NA	1357/988	NA	1404/1256	NA	749/1325	NA
**Females**								
1 Partner in past 5 y	60.5 (58.9-62.0)	NA	31.1 (28.6-33.8)	NA	61.9 (59.6-64.1)	NA	79.9 (77.4-82.2)	NA
2 Partners in past 5 y	14.8 (13.8-15.8)	100	18.7 (16.8-20.8)	100	15.8 (14.1-17.5)	100	11.1 (9.4-13.0)	100
Time overlap (concurrency)	4.0 (3.4-4.6)	27.0	3.5 (2.7-4.5)	18.5	4.7 (3.8-5.7)	29.8	3.7 (2.7-5.0)	33.3
Time gap (serially monogamous)	10.1 (9.3-11.0)	68.3	13.9 (12.2-15.7)	74.1	10.4 (9.1-11.9)	66.0	7.2 (5.8-8.8)	64.9
Timing unknown	0.7 (0.5-1.0)	4.7	1.4 (0.8-2.3)	7.4	0.7 (0.4-1.2)	4.2	0.2 (0.1-0.8)	1.9
3 Partners in past 5 y	24.8 (23.4-26.2)	100	50.2 (47.5-52.9)	100	22.4 (20.5-24.4)	100	9.1 (7.5-11.0)	100
Both time overlaps (concurrent)	2.0 (1.6-2.4)	7.9	3.1 (2.2-4.3)	6.1	1.7 (1.2-2.3)	7.5	1.5 (0.9-2.3)	16.0
Both time gaps (serially monogamous)	11.2 (10.2-12.2)	45.0	24.6 (22.3-27.0)	49.0	9.7 (8.4-11.2)	43.4	3.0 (2.2-4.1)	33.2
1 Time overlap (concurrent), 1 time gap (serially monogamous)	7.9 (7.2-8.7)	32.0	14.2 (12.5-16.0)	28.3	8.2 (7.0-9.5)	36.6	3.3 (2.3-4.6)	36.1
Timing unknown	3.7 (3.1-4.3)	15.0	8.3 (7.0-9.9)	16.6	2.8 (2.2-3.6)	12.5	1.3 (0.7-2.5)	14.8
Any concurrency^b^	13.9 (12.9-15.0)	NA	20.7 (18.7-22.9)	NA	14.7 (13.1-16.2)	NA	8.4 (6.9-10.2)	NA
Denominators, unweighted/weighted	5158/3585	NA	1717/955	NA	2318/1281	NA	1123/1349	NA

Abbreviation: NA, not applicable.

^a^ Data are from the third National Survey of Sexual Attitudes and Lifestyles, a stratified probability sample survey of 15 162 people aged 16 to 74 years among residents in Britain. Denominators were defined as participants reporting 1 or more most recent partners in the 5 years before the interview and 1 or more most recent partners in the most recent partner module. The percentages (95% CIs) are based on weighted data.

^b^ Excludes most recent partners for which the timing is unknown.

Partnership histories varied by age group, but these variations were similar for males and females. The proportion of participants reporting multiple most recent partners (and thus the potential for overlapping partnerships) decreased with age. For example, 56.9% of males aged 16 to 24 years reported 3 most recent partners compared with 33.5% of males aged 25 to 34 years, and 15.3% of males aged 35 to 44 years. However, among those who reported multiple most recent partners, the proportion who had these partners concurrently increased with age. For example, of males reporting 2 most recent partners, 12.5% of those aged 16 to 24 years had these partnerships concurrently compared with 39.4% of those aged 34 to 44 years.

### Distribution of Time Gaps Between Partners

**Figure 1.**
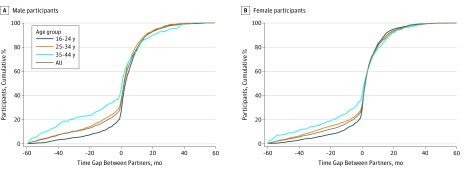
Cumulative Percentage Distribution of Time Gaps Between Partners for Sexually Active Males and Females Aged 16 to 44 Years in Britain Reporting 2 or More Most Recent Partners by Age Group Data are from the third National Survey of Sexual Attitudes and Lifestyles, a stratified probability sample survey of 15 162 people aged 16 to 74 years among residents in Britain that was undertaken from 2010 to 2012. The denominator was the time gaps between the most recent partner and second most recent partner, and where applicable, between the second and third most recent partners among 1967 males (weighted, 1651) and 2373 females (weighted, 1395).

Figure 1 shows the cumulative distribution of all time gaps between partners (n = 7094) reported by participants with 2 or more most recent partners among 1967 (weighted, 1651) males and 2373 (weighted, 1395) females. The median time gap between males’ partners was 2 months (IQR, −2 months to 9 months) and between females’ partners was 2 months (−1 month to 8 months), indicating that the majority of males and females reporting multiple partners had been serially monogamous. Overall, 67.0% of all time gaps were of 1 month or more.

Differences in the distribution of time gaps were observed by age group. Of note, the lower quartile was increasingly negative with increasing age, indicating longer periods of concurrency: for males, 0-month time gap for ages 16 to 24 years, −5-month time gap for age 25 to 34 years, and −17-month time gap for age 35 to 44 years; for females, 0-month time gap for age 16 to 24 years, −2-month time gap for age 25 to 34 years, and −9-month time gap for age 35 to 44 years.

### Perceptions of Most Recent Partners’ Concurrency

Around one-quarter of participants reporting 1 or more most recent partner responded yes to the question of whether they thought that any of their 3 most recent partners had had sex with other partners since they first had sex together (29.9% of male participants; 27.9% of female participants), whereas an additional 9.8% responded probably (Table 2). Females were more likely than males to say with certainty that none of their most recent partners had had sex with concurrent partners (54.7% vs 50.7%, *P* = .003). Table 2 also shows that as the number of most recent partners reported increased, the proportion of male and female participants who thought that their most recent partner had had sex with concurrent partners also increased.

**Table 2.** Participant Perception of Most Recent Partner Concurrency by Participant Sex and Number of Most Recent Partners Reported Among Sexually Active Participants Aged 16 to 44 Years in Britain, 2010-2012^a^

Variable	Participant Response to Perceived Most Recent Partner Having Had Sex With Others Since the First Sexual Encounter With the Participant, % (95% CI)^b^				Denominators, Unweighted/Weighted^c^
	Yes	Probably	Probably Not	No	
**Males**					
Any of the most recent partners	29.9 (28.1-31.8)	9.8 (8.7-11.0)	9.6 (8.4-10.8)	50.7 (48.6-52.8)	3238/3337
1 Partner	15.2 (13.2-17.5)	4.0 (2.9-5.4)	5.5 (4.2-7.1)	75.3 (72.6-77.9)	1352/1698
2 Partners					
Most recent partner	21.2 (17.0-26.1)	7.5 (5.2-10.7)	13.2 (9.9-17.4)	58.1 (52.8-63.3)	462/457
Second most recent partner	26.2 (21.3-31.8)	17.4 (13.1-22.6)	17.5 (13.1-22.9)	39.0 (33.6-44.7)	377/359
3 Partners					
Most recent partner	26.6 (23.7-29.7)	9.0 (7.2-11.1)	11.9 (9.9-14.3)	52.5 (49.1-55.9)	1156/972
Second most recent partner	34.4 (30.8-38.1)	19.2 (16.3-22.5)	14.8 (12.2-17.8)	31.6 (28.2-35.3)	869/713
Third most recent partner	35.6 (31.8-39.5)	14.3 (11.8-17.3)	15.9 (13.1-19.3)	34.2 (30.7-38.0)	848/699
**Females**					
Any of the most recent partners	27.9 (26.5-29.4)	9.8 (8.9-10.8)	7.6 (6.8-8.5)	54.7 (53.1-56.3)	4940/3343
1 Partner	17.3 (15.8-19.0)	4.8 (3.9-5.8)	4.6 (3.7-5.5)	73.3 (71.3-75.2)	2584/2061
2 Partners					
Most recent partner	20.9 (17.8-24.4)	8.4 (6.3-11.3)	9.5 (7.4-12.1)	61.2 (57.0-65.2)	784/476
Second most recent partner	28.0 (24.1-32.3)	17.3 (14.1-21.0)	13.2 (10.2-17.0)	41.5 (37.0-46.2)	648/399
3 Partners					
Most recent partner	23.6 (21.2-26.3)	7.2 (5.8-9.0)	8.5 (6.9-10.5)	60.7 (57.5-63.7)	1327/765
Second most recent partner	32.8 (29.7-36.0)	17.4 (14.9-20.2)	13.5 (11.2-16.2)	36.3 (32.9-39.8)	1044/594
Third most recent partner	34.1 (30.8-37.6)	18.2 (15.7-21.0)	11.6 (9.6-14.0)	36.1 (32.9-39.5)	1022/590

^a^ Data are from the third National Survey of Sexual Attitudes and Lifestyles, a stratified probability sample survey of 15 162 people aged 16 to 74 years among residents in Britain.

^b^ Excludes partnerships in which sex only occurred once because the question was not asked about such partners.

^c^ Denominators were defined as participants reporting 1 or more partners in the 5 years before the interview and 1 or more most recent partners in the most recent partner module. Denominators for each of the partner categories do not sum to the denominator for “any of the most recent partners” owing to missing data for each most recent partner number. The percentages (95% CIs) are based on weighted data. Priority was given to a response of yes for any of the 3 most recent partners, followed by probably and probably not, with no meaning that the participant did not think that any of their 3 most recent partners had any concurrent partners.

### Variations in STI Diagnosis According to the Time Gap Between Most Recent Partners and Perception of Most Recent Partner Concurrency

**Figure 2.**
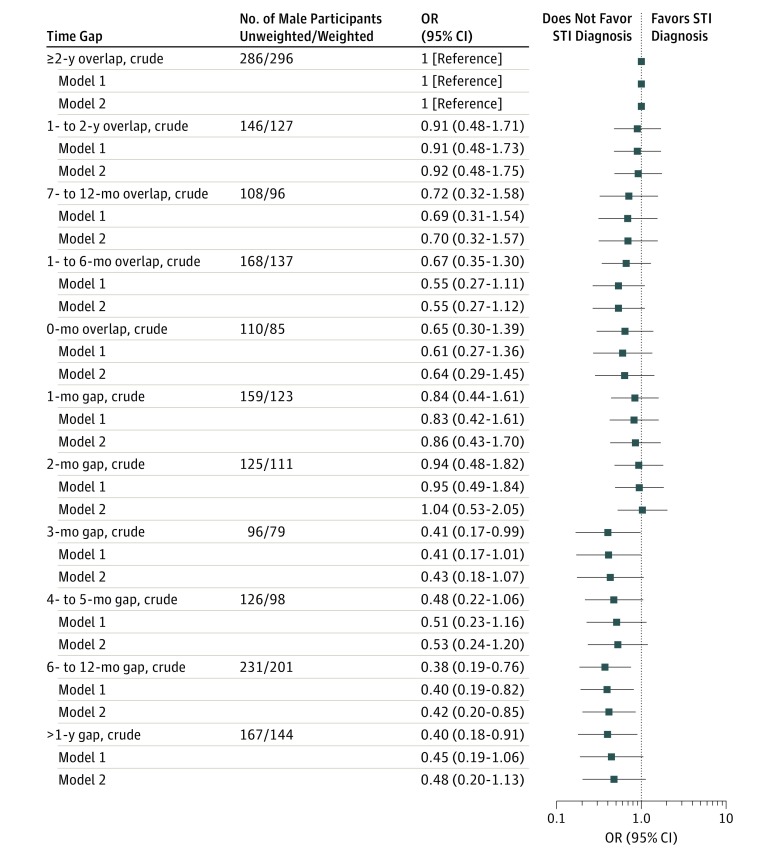
Crude and Adjusted Odds Ratios (ORs) of Males Reporting Sexually Transmitted Infection (STI) Diagnosis According to the Length of the Time Gap Between Their Most Recent Partners Data are from the third National Survey of Sexual Attitudes and Lifestyles, a stratified probability sample survey of 15 162 people aged 16 to 74 years among residents in Britain that was undertaken from 2010 to 2012. The reference category was 2 years or more time overlap (ie, partnerships overlapping in time for ≥2 years). Adjusted model 1 adjusts for age and number of partners (not capped at 3 partners) in the past 5 years. Adjusted model 2 adjusts for age, number of partners (not capped at 3 partners) in the past 5 years, and whether the participant perceived their most recent partner to have had sex with other partners since they first had sex together. The denominator was participants with data available on at least 1 time gap between most recent partners and thus was limited to participants reporting at least 2 most recent partners in the most recent partner module (1967 males; weighted, 1651). The more negative time gap (or time overlap) is used if more than 1 time gap is available (ie, for those reporting 3 most recent partners).

**Figure 3.**
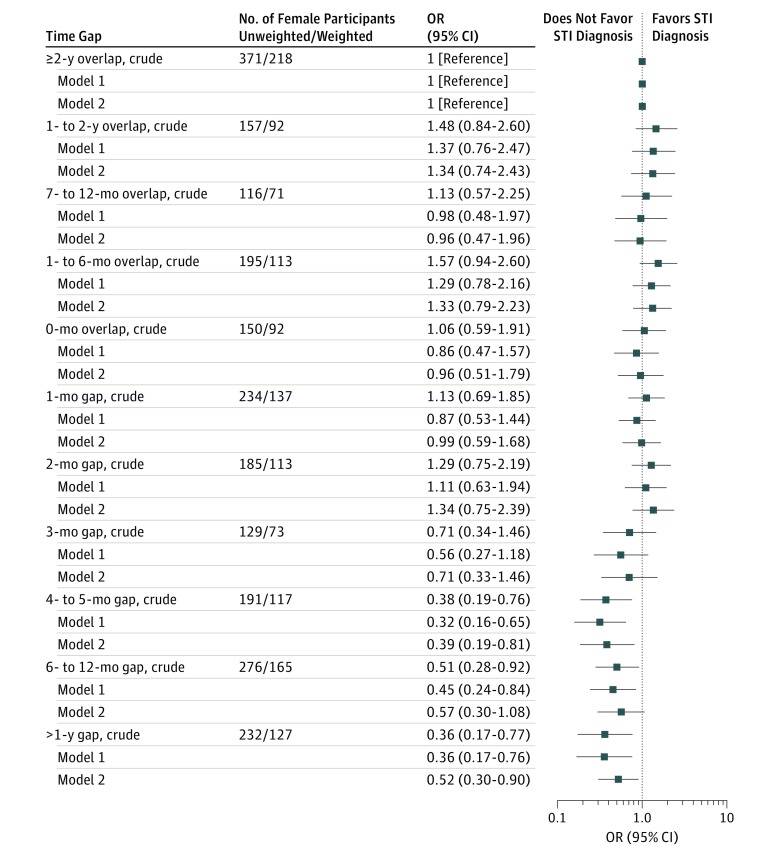
Crude and Adjusted Odds Ratios (ORs) of Females Reporting Sexually Transmitted Infection (STI) Diagnosis According to the Length of the Time Gap Between Their Most Recent Partners Data are from the third National Survey of Sexual Attitudes and Lifestyles, a stratified probability sample survey of 15 162 people aged 16 to 74 years among residents in Britain that was undertaken from 2010 to 2012. The reference category was 2 years or more time overlap (ie, partnerships overlapping in time for ≥2 years). Adjusted model 1 adjusts for age and number of partners (not capped at 3 partners) in the past 5 years. Adjusted model 2 adjusts for age, number of partners (not capped at 3) in the past 5 years, and whether the participant perceived their most recent partner to have had sex with other partners since they first had sex together. The denominator was participants with data available on at least 1 time gap between most recent partners and thus were limited to participants reporting at least 2 most recent partners in the most recent partner module (2373 women; weighted, 1395). The more negative time gap (or time overlap) is used if more than 1 time gap is available (ie, for those reporting 3 most recent partners).

Figure 2 and Figure 3 show the odds of STI diagnosis in the past 5 years for participants reporting 1 or more time gaps during that period according to the length of the time gap, with 2 years or more overlap (ie, partnership concurrency of ≥2 years) as the reference category. There was some evidence for males that an STI diagnosis was more common among those with shorter time gaps and overlaps, for example, 15.4% (95% CI, 11.1%-21.0%) of males with an overlap of 2 years or more reported an STI diagnosis in contrast to 7.1% (95% CI, 3.8%-12.9%) of males with a gap of more than 1 year; however, the overall association was not significant (*P* = .08). A similar pattern was observed for females: 13.9% (95% CI, 10.5%-18.1%) of females with an overlap of 2 years or more reported an STI diagnosis and 6.3% (95% CI, 3.5%-11.2%) of females with a gap of more than 1 year, but the overall association was significant for females (*P* < .001) (eTable 2 in the Supplement). From this analysis, we estimated the time gap (in months) between most recent partners that was associated with a significant reduction in the odds of STI diagnosis (compared with a ≥2-year time overlap). For males, this time gap was 6 months or more, whereas for females, it was 4 months or more. Adjusting for the participant’s age and number of partners (not just most recent partners [ie, not capped at 3]) had little effect on the magnitude of the odds ratios (ORs) (Figure 2 and Figure 3).

Perception of most recent partner concurrency was also associated with STI diagnosis for both males and females (eTable 3 in the Supplement), with STI diagnosis less likely among those who responded no to their partners having had sex with other partners (crude OR for male participants, 0.53; 95% CI, 0.35-0.82) compared with those responding yes (crude OR for female participants, 0.35; 95% CI, 0.24-0.52). This association remained significant for both sexes after adjusting for the participant age and number of partners (not just most recent partners [ie, not capped at 3]) (male participants responding no: adjusted OR, 0.58 [95% CI, 0.37-0.92]; female participants responding no: adjusted OR, 0.32 [95% CI, 0.22-0.49] compared with males and females reporting yes, respectively).

An investigation of whether the participant’s own concurrency (in terms of the time gap between their most recent partners) and their perception of their most recent partners’ concurrency were independently associated with STI diagnosis was performed after additionally adjusting for age and number of partners (not just most recent partners [ie, not capped at 3]) (Figure 2 and Figure 3). Neither measure remained independently associated for males (although the adjusted OR for having a time gap of 6-12 months remained significantly less than 1; adjusted OR, 0.40 [95% CI, 0.19-0.82]). For females, however, there remained evidence of both markers (the participant’s own concurrency [in terms of the time gap between their most recent partners] and the participant’s perception of their partners’ most recent partners’ concurrency) being associated with the likelihood of STI diagnosis. Adjustment had little effect on the magnitude of the adjusted ORs, with these continuing to show a reduced likelihood of STI diagnosis compared with reference categories (adjusted ORs: 0.48 [95% CI, 0.29-0.79] for gaps of 4 months or more and 0.32 [95% CI, 0.22-0.49] for reporting that the participants’ most recent partners had not had sex with other partners) (eTable 2 in the Supplement).

## Discussion

### Principal Findings

Using national probability survey data, this study revealed that 48.1% of sexually active males and 39.5% of females aged 16 to 44 years in Britain had multiple sexual partners in the 5 years before the interview, and most were serially monogamous. Although recent partnership histories and the duration of the time gap between most recent partners varied by participant sex and age group, overall the time gap was short, typically a few months. Given the long infectious period of some STIs, this outcome means that biological opportunities for STI transmission may exist even among individuals who have not had concurrent partnerships.^5,6^ This study found that compared with participants whose partnerships had overlapped for 2 years or more, the likelihood of an STI diagnosis in the past 5 years did not decrease significantly until the time gap between most recent partners was 4 months or more for females and 6 months or more for males. Furthermore, this association was independent of participant age and the number of partners (not just most recent partners [ie, not capped at 3]) reported. This study also found that around one-third of the participants (male, 39.7%; female, 37.7%) knew or suspected that their partners had had sex with other partners concurrently, which was associated with a greater likelihood of STI diagnosis independent of the time gap they had had between their most recent partners, their age, and their number of partners (not just most recent partners [ie, not capped at 3]). To our knowledge, this is the first time that these questions have been addressed, at least for the British population.

### Findings in Comparison With the Existing Literature

It is difficult to make meaningful comparisons between these findings and other studies because of methodological differences in study populations and time frames, even among the relatively few studies that used the time gap to assess whether concurrency occurred.^3,23,24,25,26^ Nonetheless, a similarity is that the average time gaps were typically positive (reflecting serial monogamy) although often shorter than the mean duration of the infectious period for many bacterial STIs.^5,6^

This study’s finding that the time gap was shorter when participants perceived their most recent partners to have had sex with other partners is consistent with other studies^27,28^ and with the broader finding that individuals who have concurrent partnerships do so with those who are also likely to have other partners.^11,28^ Findings are mixed regarding the importance of an individual’s own concurrency for STI risk compared with that of their partner’s concurrency.^10,27^ This study found that perceptions about most recent partner concurrency was associated with the likelihood of STI diagnosis among females but not among males, independent of their own concurrency, age, and number of partners (not just most recent partners [ie, not capped at 3]). This sex difference could suggest that females are more likely to know, or at least suspect, that a partner has other partners compared with men, although perceptions have been shown to poorly align with partners’ actual behavior.^8,9,12^ It is also plausible that, as condom use tends to wane quickly after a first sexual encounter with an individual,^29^ reintroducing their use into a relationship could be taken to indicate a lack of trust, creating difficulties within the relationship. However, this study was unable to test this hypothesis because Natsal-3 did not collect data on condom use over time with most recent partners.

### Study Implications, Unanswered Questions, and Future Research

These analyses revealed that studying partnership- and partner-specific factors may inform the understanding of an individual’s STI risk. The findings suggest a need for measures that capture how partners and sexual networks affect an individual’s risk of STI transmission^7,8,9^; research is needed to hone the measurement of these concepts.^17^ Nonetheless, the realities of obtaining partner-reported data remain challenging, such that novel, indirect yet reliable methods (ie, reported by the individual) are needed. In this respect, qualitative research has shown that if a study’s scientific rationale is properly explained, participants are generally willing to answer questions about their sex lives and partners.^30^ A separate issue relates to the extent to which individuals are able to provide such data, especially if the recall period is long or they have had many partners, partnerships of shorter duration, or more casual partnerships, all of which are part of the epidemiological puzzle. Techniques such as using calendars and follow-up questions for clarification have been shown to help.^14,24^

The findings suggest that not only the number of partners but also the timing of partners should be taken into account to challenge the commonly held assumption that STI transmission cannot occur if partners are serially monogamous.^28,31^ Even time gaps of several months may not be sufficient to reduce the likelihood of infection. Public health practitioners seeking to develop and deliver effective STI prevention interventions can play a role in this respect. For example, the traditional focus of sexual health promotion (ie, encouraging STI testing before condomless sex with a new partner) could be made more effective if linked to the concept of the infectious period and how this can extend beyond the time gap between partners. Similarly, there is a need to communicate how STI risk and the need for STI testing are determined by not only an individual’s behaviors and characteristics but also those of their partners and their partners’ partners. These points are particularly salient given the large proportion of the population who report monogamy yet know or suspect that their partner has had sex with other partners and especially if condoms are not being used because this increases the STI risk associated with their partners’ behavior.

### Strengths and Limitations

Because data from Natsal-3 were used, these findings can be considered as broadly representative of the British general population,^14,15^ in contrast with studies that have recruited people attending sexual health clinics.^8,10,12,23,27,32,33,34^ Although most sexual behavior studies rely on reported behavior, the use of CASI in Natsal-3 encouraged accurate reporting^35,36^ of what many regard as a particularly sensitive behavior given that British society largely disapproves of concurrency, at least when 1 or more of the individuals involved are married.^15^ To reduce social desirability bias further, this study used participants’ reports of the dates of first sexual encounter and last sexual encounter with partners rather than data from a direct question about their experience of concurrency. This approach is consistent with the Joint United Nations Programme on HIV and AIDS protocol for measuring concurrency.^4^ Nonetheless, inaccuracies in reporting and refusal to answer the question are unavoidable and may be more likely if participants are seeking to avoid disclosing overlapping partnerships. However, it is recognized that for some people, nonmonogamy is the norm and is not necessarily regarded as stigmatizing.^37^

Considering negative time gaps (time overlaps) and positive time gaps between partners as a continuous variable allowed for the investigation of the opportunity for STI transmission in a more nuanced way than is typically possible with the conventional dichotomous measure of concurrency. Assumptions were necessarily made about concurrency based on month of first sexual encounter, but it is unlikely that capturing dates in this way affected the measure’s sensitivity. Additionally requesting the day of first and last sexual encounters would have increased participant burden, potentially increasing item nonresponse and recall bias,^32^ especially because Natsal-3 asked about multiple partners over a fairly long period.^14^ Focusing on the 5 years before the interview in contrast to, for example, the past year enabled the study to maximize the number of partners and time gaps and thus its statistical power; this strategy was a necessity because of the low rates of partner change in the British general population.^15^ Although most participants aged 16 to 44 years reported 3 or fewer partners in the previous 5 years, 29% of males and 20% of females reported 4 or more partners (ie, additional partners about whom the Natsal-3 most recent partner module did not ask to minimize participant burden). Because larger numbers of most recent partners were associated with shorter time gaps, this study was likely to have underestimated concurrency prevalence in contrast with asking a direct question about any concurrency throughout the time frame, as others studies have found.^24,32,38^ It is also likely that for those with 4 or more partners, the outcome of interest, STI diagnosis in the past 5 years, may not have resulted from 1 of their 3 or fewer most recent partners but from a higher-order (ie, earlier) partner. Also, we were unable to capture undiagnosed STIs and only captured STIs diagnosed and reported by participants. In this analysis of how reporting STI diagnosis varies by the length of the time gap, we sought to balance the categorization of the time gap so that it was sufficiently nuanced to be meaningful yet pragmatic in terms of sample size; however, although the subgroups were a similar size, some nonsignificant time gaps could reflect a lack of statistical power.

Focusing on 3 or fewer most recent partners meant that for some participants, the study was unable to capture their perceptions of all their most recent partners’ concurrency, and thus it was necessary to assume that these partners were representative of all their recent partners. These limitations may be of greater significance for particular population groups who tend to have larger numbers of partners (eg, men who have sex with men),^39^ but the aim of this study was to consider the general population perspective and these groups constitute a relatively small proportion of the total population.^15^ It is unlikely that the inclusion or exclusion of these groups would substantially alter the population estimates generated by this study. The wording of the question about participant perception of most recent partners’ concurrency depended on whether the partnership had ended,^14^ and if so, it is plausible that some recasting occurred because of the knowledge gained since the partnership ended. More broadly, it is unlikely that the limited, quantitative data analyzed can truly capture the complexities of partnership formation or dissolution.

## Conclusions

Among sexually active people aged 16 to 44 years in Britain, STI diagnosis was associated with timing of partners for both males and females. The participant perception of a partners’ time overlap (concurrency) with another partner was also associated with STI diagnosis among females only. This study suggests the epidemiological importance of understanding the timing of partners in STI transmission, not just the timing of an individual’s partners but also of their partners’ partners.

## Notes

Supplement. **eFigure.** Examples of Most Recent Partnership (MRP) Timings, Concurrent and Serially Monogamous Partnerships, and How the Gap Is Calculated **eTable 1.** Characteristics of Study Sample, by Sex **eTable 2.** Variation in the Reporting of Sexually Transmitted Infection Diagnosis/es in the Past Five Years According to the Gap Between Participants’ Most Recent Partners, by Sex **eTable 3.** Association Between Participants’ Perceptions of Their Recent Partners’ Concurrency and Sexually Transmitted Infection Diagnosis by Sex
