# Supplementary material for: Association of Timing of Sexual Partnerships and Perceptions of Partners' Concurrency With Reporting of Sexually Transmitted Infection Diagnosis
Source: JAMA Netw Open. 2018 Dec 14;1(8):e185957. doi: 10.1001/jamanetworkopen.2018.5957 (PMC6324336; doi:10.1001/jamanetworkopen.2018.5957)
Supplement: Supplement. — eFigure. Examples of Most Recent Partnership (MRP) Timings, Concurrent and Serially Monogamous Partnerships, and How the Gap Is Calculated eTable 1. Characteristics of Study Sample, by Sex eTable 2. Variation in the Reporting of Sexually Transmitted Infection Diagnosis/es in the Past Five Years According to the Gap Between Participants’ Most Recent Partners, by Sex eTable 3. Association Between Participants’ Perceptions of Their Recent Partners’ Concurrency and Sexually Transmitted Infection Diagnosis by Sex [file jamanetwopen-1-e185957-s001.pdf]

Supplementary Online Content

Mercer CH, Jones KG, Geary RS, et al. Association of sexual partners' timing and perceptions of partners' concurrency with reporting of sexually transmitted infection diagnosis. *JAMA Netw Open*. 2018;1(8):e185957. doi:10.1001/jamanetworkopen.2018.5957

eFigure. Examples of Most Recent Partnership (MRP) Timings, Concurrent and Serially Monogamous Partnerships, and How the Gap Is Calculated

eTable 1. Characteristics of Study Sample, by Sex

eTable 2. Variation in the Reporting of Sexually Transmitted Infection Diagnosis/es in the Past Five Years According to the Gap Between Participants' Recent Partners, by Sex

eTable 3. Association Between Participants' Perceptions of Their Recent Partners' Concurrency and Sexually Transmitted Infection Diagnosis by Sex

This supplementary material has been provided by the authors to give readers additional information about their work.

eFigure. Examples of Most Recent Partnership (MRP) Timings, Concurrent and Serially Monogamous Partnerships, and How the Gap Is Calculated

Date (month, year)														
Jan-11	Feb-11	Mar-11	Apr-11	May-11	Jun-11	Jul-11	Aug-11	Sep-11	Oct-11	Nov-11	Dec-11	Jan-12	Feb-12	Mar-12
						E	MRP							F
						Overlap of 4-mo. (so gap = -4-mo.)**								
					C	2nd MRP			D					
			+2-mo. gap*											
A	3rd MRP	B												

Time point:
A – month/year of first sex with 3rd most recent partner (January 2011)
B – month/year of last sex with 3rd most recent partner (March 2011)
C – month/year of first sex with 2nd most recent partner (June 2011)
D – month/year of last sex with 2nd most recent partner (October 2011)
E – month/year of first sex with most recent partner (July 2011)
F – month/year of last sex with most recent partner (March 2012)

* Gap of 2 months between last sex with 3rd most recent partner and first sex with 2nd most recent partner meaning that the 3rd and 2nd most recent partners were serially monogamous.

** Overlap of 4 months between last sex with 2nd most recent partner and first sex with most recent partner meaning that the 2nd most recent partner and most recent partner were concurrent for 4 months. Using gap terminology, an overlap of 4 months is labelled as a gap of -4 months.

© Mercer CH et al. *JAMA Network Open*.

eTable 1. Characteristics of Study Sample, by Sex

	**All men aged 16-44** % (95% CI)	**All women aged 16-44** % (95% CI)
**Age, grouped**		
16-24	27.7% (26.0-29.4)	26.6% (25.4-27.9)
25-34	35.2% (33.4-37.1)	35.7% (34.3-37.2)
35-44	37.1% (35.0-39.3)	37.6% (36.0-39.3)
**Relationship status at interview**		
Living with a partner	58.9% (56.9-60.9)	62.5% (60.9-63.9)
In a steady relationship (but not living together)	16.7% (15.4-18.1)	16.8% (15.8-17.8)
Not in steady relationship	24.4% (22.9-26.0)	20.8% (19.5-22.1)
**Sexual identity**		
Heterosexual/straight	96.9% (96.1-97.6)	96.0% (95.4-96.6)
Gay/lesbian	1.9% (1.4-2.5)	1.3% (1.0-1.8)
Bisexual	1.0% (0.6-1.6)	2.4% (2.0-2.9)
Other	0.2% (0.1-0.3)	0.3% (0.1-0.5)
**Ethnic group**		
White/white British	85.7% (84.1-87.2)	86.8% (85.6-87.9)
Asian/Asian British	6.8% (5.7-8.1)	5.7% (4.9-6.6)
Black/Black British	3.7% (3.0-4.6)	3.7% (3.1-4.4)
Mixed ethnicity	2.3% (1.8-2.9)	2.6% (2.2-3.1)
Other ethnicity	1.5% (1.0-2.1)	1.3% (1.0-1.7)
**Religious affiliation**		
None	62.3% (60.2-64.3)	54.1% (52.4-55.7)
Christian	29.8% (28.0-31.7)	39.3% (37.7-40.9)
Muslim	4.0% (3.3-4.9)	3.6% (3.0-4.4)
Hindu	2.0% (1.4-3.0)	1.2% (0.9-1.5)
Other non-Christian	1.9% (1.4-2.5)	1.9% (1.5-2.4)
**Academic qualifications**[2]		
None	9.9% (8.7-11.3)	7.9% (7.1-8.9)
Academic qualifications typically gained at age 16[3]	36.0% (34.1-38.0)	35.5% (33.9-37.2)
Studying for/attained further academic qualifications	54.1% (51.9-56.2)	56.6% (54.8-58.3)
**National Statistics Socio-Economic Classification**[4]		
Managerial/professional occupations	34.7% (32.7-36.6)	32.7% (31.2-34.3)
Intermediate occupations	15.0% (13.5-16.5)	19.8% (18.6-21.2)
Semi-routine/routine occupations	35.2% (33.3-37.1)	27.7% (26.4-29.2)
No job (for 10+ hours/week)	2.6% (2.1-3.3)	7.6% (6.7-8.5)
Student in full-time education	12.6% (11.3-14.0)	12.1% (11.1-13.2)
**Quintile of Adjusted Index of Multiple Deprivation**[5]		
I (least deprived)	18.1% (16.4-20.0)	17.4% (15.9-19.0)
II	19.3% (17.6-21.1)	19.3% (17.8-20.9)
III	19.6% (17.9-21.5)	19.9% (18.3-21.6)
IV	22.4% (20.5-24.4)	22.2% (20.7-23.9)
V (most deprived)	20.6% (18.8-22.5)	21.2% (19.6-22.9)
Denominators[1,6]	*3509, 3568*	*5158, 3585*

Notes for eTable 1:

1. Defined as participants reporting ≥1 sexual partner(s) in the five years prior to interview and ≥1 partner(s) in the MRP module.
2. Participants aged ≥17 years.
3. English General Certificate of Secondary Education or equivalent.
4. As per the National Statistics Socio-Economic Classification.[21]
5. An area-level measure of deprivation.[22]
6. Unweighted, weighted denominators. % (95% confidence interval, CI) are based on weighted data.

© Mercer CH et al. *JAMA Network Open*.

eTable 2. Variation in the Reporting of Sexually Transmitted Infection Diagnosis/es in the Past Five Years According to the Gap Between Participants' Recent Partners, by Sex

	% (95% CI) reporting STI diagnosis	Crude odds ratio (95% CI)	Adjusted odds ratio[1] (95% CI)	Adjusted odds ratio[2] (95% CI)	*Denominators*[3]
**Men**					
**All**	**11.2% (9.6-13.0)**	-	-	-	*1719, 1497*
**By gap width:**					
≥2yr overlap	15.4% (11.1-21.0)	1.00	1.00	1.00	*286, 296*
1-2yr overlap	14.2% (9.1-21.5)	0.91 (0.48, 1.71)	0.91 (0.48, 1.73)	0.92 (0.48, 1.75)	*146, 127*
7-12-mo. overlap	11.5% (6.1-20.7)	0.72 (0.32, 1.58)	0.69 (0.31, 1.54)	0.70 (0.32, 1.57)	*108, 96*
1-6-mo. overlap	10.9% (6.6-17.4)	0.67 (0.35, 1.30)	0.55 (0.27, 1.11)	0.55 (0.27, 1.12)	*168, 137*
0-mo. overlap	10.5% (5.7-18.7)	0.65 (0.30, 1.39)	0.61 (0.27, 1.36)	0.64 (0.29, 1.45)	*110, 85*
1-mo. gap	13.3% (8.2-20.9)	0.84 (0.44, 1.61)	0.83 (0.42, 1.61)	0.86 (0.43, 1.70)	*159, 123*
2-mo. gap	14.6% (9.0-22.8)	0.94 (0.48, 1.82)	0.95 (0.49, 1.84)	1.04 (0.53, 2.05)	*125, 111*
3-mo. gap	6.9% (3.2-14.3)	0.41 (0.17, 0.99)	0.41 (0.17, 1.01)	0.43 (0.18, 1.07)	*96, 79*
4-5-mo. gap	8.0% (4.1-15.1)	0.48 (0.22, 1.06)	0.51 (0.23, 1.16)	0.53 (0.24, 1.20)	*126, 98*
6-12-mo. gap	5.9% (3.4-10.1)	0.38 (0.19, 0.76)	0.40 (0.19, 0.82)	0.42 (0.20, 0.85)	*231, 201*
>1 yr gap	7.1% (3.8-12.9)	0.40 (0.18, 0.91)	0.45 (0.19, 1.06)	0.48 (0.20, 1.13)	*167, 144*
**Women**					
**All**	**12.8% (11.3-14.5)**	-	-	-	*2236, 1317*
**By gap width:**					
≥2yr overlap	13.9% (10.5-18.1)	1.00	1.00	1.00	*371, 218*
1-2yr overlap	19.2% (13.1-27.4)	1.48 (0.84, 2.60)	1.37 (0.76, 2.47)	1.34 (0.74, 2.43)	*157, 92*
7-12-mo. overlap	15.4% (9.0-25.1)	1.13 (0.57, 2.25)	0.98 (0.48, 1.97)	0.96 (0.47, 1.96)	*116, 71*
1-6-mo. overlap	20.2% (14.5-27.4)	1.57 (0.94, 2.60)	1.29 (0.78, 2.16)	1.33 (0.79, 2.23)	*195, 113*
0-mo. overlap	14.6% (9.4-21.9)	1.06 (0.59, 1.91)	0.86 (0.47, 1.57)	0.96 (0.51, 1.79)	*150, 92*
1-mo. gap	15.4% (11.1-21.0)	1.13 (0.69, 1.85)	0.87 (0.53, 1.44)	0.99 (0.59, 1.68)	*234, 137*
2-mo. gap	17.2% (11.7-24.5)	1.29 (0.75, 2.19)	1.11 (0.63, 1.94)	1.34 (0.75, 2.39)	*185, 113*
3-mo. gap	10.2% (5.6-17.9)	0.71 (0.34, 1.46)	0.56 (0.27, 1.18)	0.71 (0.33, 1.52)	*129, 73*
4-5-mo. gap	5.7% (3.1-10.4)	0.38 (0.19, 0.76)	0.32 (0.16, 0.65)	0.39 (0.19, 0.81)	*191, 117*
6-12-mo. gap	7.2% (4.5-11.4)	0.51 (0.28, 0.92)	0.45 (0.24, 0.84)	0.57 (0.30, 1.08)	*276, 165*
>1 yr gap	6.3% (3.5-11.2)	0.36 (0.17, 0.77)	0.36 (0.17, 0.76)	0.52 (0.30, 0.90)	*232, 127*

© Mercer CH et al. *JAMA Network Open*.

Notes for eTable 2:

1. Adjusted odds ratios from adjusting for age and partner numbers (past five years).
2. Adjusted odds ratios from adjusting for age, partner numbers (past five years) and whether the participant perceived their partner to have had other partners since they first had sex together.
3. Unweighted/weighted denominators, defined as participants reporting ≥2 partner(s) in the MRP module without missing data for the variables included in the multivariable models. % (95% CI) are based on weighted data.

© Mercer CH et al. *JAMA Network Open*.

eTable 3. Association Between Participants' Perceptions of Their Recent Partners' Concurrency and Sexually Transmitted Infection Diagnosis by Sex[1]

Perceived Any Recent Partner to Have Had Other Partner(s) Since They First Had Sex Together[2,3]	STI Diagnosis/es, % (95% CI)	Crude Odds Ratio (95% CI)	*P* Value	Adjusted For Participant's Age & Partner Numbers, Odds Ratio (95% CI)[1]	*P* Value	*Denominator*[4]
**Men**			.028		.089	
Yes	12.7 (10.4-15.5)	1 [Ref]		1 [Ref]		*764, 661*
Probably	13.3 (8.9-19.3)	1.05 (0.64-1.72)		1.08 (0.64-1.83)		*263, 227*
Probably not	11.5 (7.6-17.1)	0.89 (0.53-1.50)		0.93 (0.55-1.56)		*233, 210*
No	7.2 (5.1-10.1)	0.53 (0.35-0.82)		0.58 (0.37-0.92)		*462, 399*
**Women**			<.001		<.001	
Yes	17.1 (14.5-20.0)	1 [Ref]		1 [Ref]		*988, 571*
Probably	11.8 (8.4-16.4)	0.65 (0.43-0.99)		0.67 (0.44-1.02)		*364, 222*
Probably not	13.2 (9.0-18.9)	0.74 (0.46-1.18)		0.77 (0.48-1.25)		*260, 159*
No	6.7 (4.9-9.2)	0.35 (0.24-0.52)		0.32 (0.22-0.49)		*624, 366*

Notes for eTable 3:

Abbreviations: MRP, most recent partner, STI, sexually transmitted infection.

1. In the five years prior to interview.
2. Defined as any of the ≤3 MRPs.
3. Excludes partnerships where sex only occurred once as question not asked about such partners.
4. Unweighted, weighted denominators being those participants sexually-active in the past five years (defined as participants reporting ≥1 sexual partner(s) in the five years prior to interview and ≥1 partner(s) in the MRP module) who reported sex with their former partner more than once. % (95% CI) are based on weighted data.

© Mercer CH et al. *JAMA Network Open*.
